# Intramuscular Inoculation of AS02-Adjuvanted Respiratory Syncytial Virus (RSV) F Subunit Vaccine Shows Better Efficiency and Safety Than Subcutaneous Inoculation in BALB/c Mice

**DOI:** 10.3389/fimmu.2022.938598

**Published:** 2022-07-22

**Authors:** Lijun Bian, Yu Zheng, Xiaohong Guo, Dongdong Li, Jingying Zhou, Linyao Jing, Yan Chen, Jingcai Lu, Ke Zhang, Chunlai Jiang, Yong Zhang, Wei Kong

**Affiliations:** ^1^ National Engineering Laboratory for AIDS Vaccine, School of Life Sciences, Jilin University, Changchun, China; ^2^ Key Laboratory for Molecular Enzymology and Engineering, The Ministry of Education, School of Life Sciences, Jilin University, Changchun, China; ^3^ NMPA Key Laboratory of Humanized Animal Models for Evaluation of Vaccines and Cell Therapy Products, Jilin University, Changchun, China; ^4^ R&D Center, Changchun BCHT Biotechnology Co., Changchun, China; ^5^ The Key and Characteristic Laboratory of Modern Pathogen Biology, Department of Parasitology, Basic Medical College, Guizhou Medical University, Guiyang, China

**Keywords:** respiratory syncytial virus, subunit vaccine, vaccine adjuvant, inoculation route, administration route

## Abstract

We previously explored a panel of adjuvants formulated with pre-fusion RSV-F protein and found that AS02 may be a promising candidate adjuvant for developing RSV-F subunit vaccines with improved immunogenicity and desired immune response type. In this study, we performed a head-to-head comparison of the effect of intramuscular injection to that of subcutaneous injection on the immune response and protective efficacy of recombinant RSV-F subunit vaccine with or without adjuvants (Alhydrogel, squalene-based emulsion adjuvants MF59, AS03, and AS02) in BALB/c mice. After inoculations, antigen-specific antibodies, neutralizing antibodies, antibody subtypes, cytokines, and the persistence of immune response were evaluated. Moreover, challenge tests were also performed to illustrate the possible effect of inoculation routes and adjuvant on virus clearance and histochemistry changes in the lungs of mice. The results indicated that intramuscular inoculation is a more effective and antigen dose-sparing route to enhance the immune response, although subcutaneous inoculation induced faster and stronger IgG antibodies after the initial immunization. Furthermore, adjuvant, but not immunization route, is a more critical factor to affect the humoral/cellular immune response and the immune bias. In addition, adjuvant inoculated *via* the intramuscular route is safer than that *via* the subcutaneous route, especially for AS02. This study highlights the importance of the adjuvant and immunization routes in the design and clinical transformation of adjuvanted vaccines. Further investigation is needed to illustrate the mechanism underlying the above difference in both efficiency and safety.

## Introduction

Respiratory syncytial virus (RSV) is a common cause of acute respiratory diseases. RSV infection occurs in people of all ages, and it can be life-threatening for infants, the elderly, and immunocompromised adults. Although many candidate vaccines are being developed, no licensed commercial vaccine has been approved yet (1). Currently, prophylactic RSV vaccines at the clinical stage include live-attenuated/chimeric, virus vector-based, protein-based, and nucleic acid vaccines (1–3). Among them, RSV-F protein-based subunit vaccines have shown better safety and promising efficacy (4), and several have entered Phase III clinical trials, such as Pfizer’s RSVpreF vaccine (NCT04424316), GSK’s RSV PreF3 (NCT04605159) and RSVPreF3 OA (NCT04886596). However, subunit vaccines are of low immunogenicity inherently and induce a short-lived immune response, usually requiring the addition of adjuvants (5, 6).

Adjuvants can modulate immune response bias, improve vaccine efficacy, save antigen dose, and reduce the number of vaccinations required to achieve adequate protective efficacy (7, 8). Adjuvants used in vaccines approved for human use and under clinical investigations are usually more advantageous in safety, manufacture, and regulation, which include aluminum salts, oil-in-water emulsion (MF59, AS03) (9, 10), AS02 (the combination of AS03 with 3’-O-deacylated monophosphoryl lipid A (MPL) and QS-21) (11), AS04 (MPL-absorbed aluminum salts) (12) and AS01 (MPL- and QS-21-loaded liposomes) (13). We previously performed head-to-head comparisons of adjuvant activities of Alhydrogel, MF59, AS03, AS02, and glycol chitosan in BALB/c mice by formulating them with pre-fusion RSV-F protein. We found that AS02-adjuvanted RSV-F subunit vaccine elicited high and long-lasting neutralizing antibody responses, robust T helper type 1 (Th1) immune response, and efficient protection after challenge (14). However, in addition to adjuvant types, other factors, such as inoculation routes, may affect the immunogenicity and safety of vaccines, and therefore their efficacy in clinical transformation (15).

In general, inoculation routes include mucosal immunization, intradermal, intramuscular (i.m.), and subcutaneous (s.c.) injections. Although preferred in clinical practice, mucosal immunization is mainly limited to live attenuated vaccines, but not adjuvanted recombinant subunit vaccines due to safety and technical challenges, such as potential immunological tolerance, short residence time, and antigen degradation, all of which lead to a low-level and short-lived immune response (16–18). Regarding intradermal immunization, it usually needs trained operators and special equipment, making it not suitable as a widely-used inoculation route, especially during epidemics and pandemics. Besides, local adverse reactions are more common after intradermal immunization (19–21). Vaccines currently approved for human use are mainly administered *via* intramuscular and subcutaneous routes. Intramuscular administration of vaccines has been reported to optimize immunogenicity, minimize adverse reactions, and achieve rapid recruitment of immune cells due to the better vascularization of muscle, and is commonly used for adjuvanted vaccines (22, 23). Benefits of subcutaneous injection include the ability to accommodate larger vaccine volumes and remain relatively uniform and slow diffuse rate, resulting in a prolonged immune response (24). Nevertheless, vaccines given *via* this route may induce different degrees of inflammatory reactions at injection sites, which is closely relevant to vaccine formulation composition, such as antigen and adjuvants (25, 26).

Despite the fact that administration routes can influence vaccine efficiency and safety to varied degrees has been confirmed in some studies, there is still no comprehensive comparison of the effect of inoculation routes on the immunogenicity and safety of adjuvanted recombinant RSV-F subunit vaccines. In this study, we attempted to compare the effect of intramuscular injection with that of subcutaneous injection on the immune response and protective efficacy of recombinant RSV-F subunit vaccine with or without adjuvants (Alhydrogel, squalene-based emulsion adjuvants MF59, AS03, and AS02) in BALB/c mice. The evaluation indexes included antigen-specific antibodies, neutralizing antibodies, antibody subtypes, and cytokines elicited by the abovementioned vaccines. Besides, the effects of inoculation routes on the persistence of immune response were evaluated up to week 16 after boosting. Moreover, challenge tests were also performed to illustrate the possible effect of the abovementioned inoculation routes on virus clearance and histochemistry changes in the lungs of mice. The aim of this study is to find a more advantageous administration route for adjuvanted recombinant RSV-F subunit vaccines from both efficiency and safety views and provide valuable data about administration routes in the design and clinical transformation of new adjuvants.

## Materials and Methods

### Cells, Virus and Vaccines

HEp-2 cells (ATCC, CCL-23) were grown in Minimum Essential Medium (MEM) supplemented with 10% fetal bovine serum and 1% Penicillin-Streptomycin. The human respiratory syncytial virus (Long strain; ATCC VR-26) was propagated in HEp-2 cells for about 5 days. Then the virus was collected, added with 25% sucrose, and freeze-thawed. After centrifugation at 8,000 rpm for 10 min, the virus solution was stored at -80°C. The RSV virus titer was determined by the median tissue culture infectious dose (TCID_50_) according to Reed-Muench assay (27).

The recombinant RSV fusion (F) protein with pre-fusion conformation was prepared as described in our previous study (14). Briefly, to keep RSV F in a pre-fusion state, the arginine residues in the two multibasic furin cleavage sites were mutated to lysine residues (RARR to KAKK and KKRKRR to KKKKKK). An artificial GCN4 isoleucine zipper trimerization motif was used to assure the trimer conformation (28, 29). Adjuvants used in this study included 2% Alhydrogel^®^ (Brenntag Biosector, Denmark) and squalene-based oil-in-water emulsions MF59, AS03, and AS02. These oil-in-water emulsion adjuvants with uniform particle size (Z-average size, 150–160 nm) were prepared *via* our vaccine adjuvant platform technology, and all PDI values of these adjuvants are less than 0.15 (30–32).

### Animal Studies

**Figure 1:**
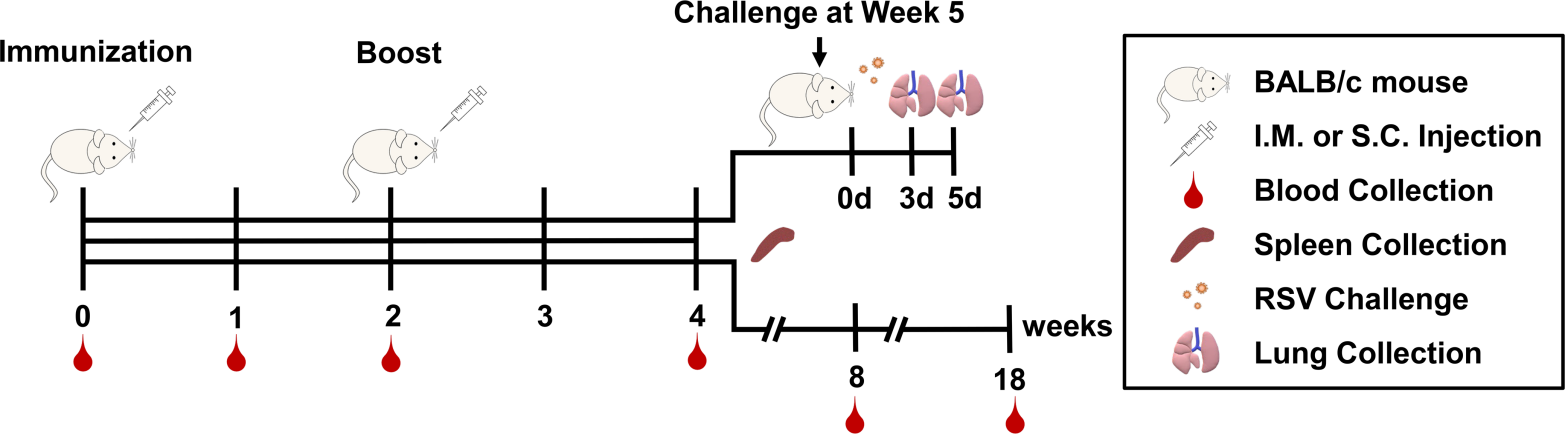
Timeline for immunization, challenge, blood, and tissue sampling schedules.

Specific-pathogen-free female BALB/c mice aged 6-8 weeks were purchased from Liaoning Changsheng Biotechnology Co. Ltd (Liaoning, China) and maintained under standard approved conditions. The mice were fed for one week after arriving in the laboratory to ensure that the mice were normal in weight and health when immunized. The experimental timeline is shown in **Figure 1** and the grouping scheme is summarized in **Table 1**. Mice were administered intramuscularly or subcutaneously with RSV-F protein alone or adjuvanted RSV-F vaccines twice at a 2-week interval. Each mouse in the intramuscular injection groups was given 50 μL vaccines (60 μL in the AS02 i.m. group), consisting of 100 µg 2% Alhydrogel^®^ in the AL i.m. group or adjuvants containing 2.15 mg squalene in squalene-based vaccine groups to ensure a consistent adjuvant dose. Besides, one dose of AS03 also contained 2.385 mg α-tocopherol, while one dose of AS02 contained 15 µg QS21 and 20 µg MPL. As for subcutaneous injection groups, the injection volume and the amount of adjuvants were twice those of intramuscularly immunized groups. PBS was used as a negative control. Bodyweight changes were monitored every week after immunization. In addition, blood samples were collected at weeks 1 and 2 after the first immunization (pre-boost), and week 2 after boosting. Then the serum was isolated by centrifugation twice at 3,000 rpm for 8 min and stored at -80 °C for antibody detection. At week 2 after boosting, five mice were sacrificed per group and spleens were harvested. Another batch of mice was used to evaluate the persistence of immune response in the intramuscular injection groups and the high-dose antigen subcutaneous injection groups. The immunization procedure was the same as above, and the blood sampling time points were weeks 2, 6, and 16 after boosting.

**Table 1:** Grouping Scheme.

Group	Treatments (2 doses; Week 0/2)	Injection volume	Inoculation route
**PBS**	PBS	50 μL	i.m.
**i.m. RSV-F**	RSV-F(1 μg)	50 μL	i.m.
**i.m. AL**	RSV-F(1 μg)/2% Alhydrogel^®^	50 μL	i.m.
**i.m. MF59**	RSV-F(1 μg)/MF59	50 μL	i.m.
**i.m. AS03**	RSV-F(1 μg)/AS03	50 μL	i.m.
**i.m. AS02**	RSV-F(1 μg)/AS02	60 μL	i.m.
**s.c. L RSV-F**	RSV-F(1 μg)	100 μL	s.c.
**s.c. L AL**	RSV-F(1 μg)/2% Alhydrogel^®^	100 μL	s.c.
**s.c. L MF59**	RSV-F(1 μg)/MF59	100 μL	s.c.
**s.c. L AS03**	RSV-F(1 μg)/AS03	100 μL	s.c.
**s.c. L AS02**	RSV-F(1 μg)/AS02	120 μL	s.c.
**s.c. H RSV-F**	RSV-F(10 μg)	100 μL	s.c.
**s.c. H AL**	RSV-F(10 μg)/2% Alhydrogel^®^	100 μL	s.c.
**s.c. H MF59**	RSV-F(10 μg)/MF59	100 μL	s.c.
**s.c. H AS03**	RSV-F(10 μg)/AS03	100 μL	s.c.
**s.c. H AS02**	RSV-F(10 μg)/AS02	120 μL	s.c.

For the RSV challenge, immunized mice were anesthetized by intraperitoneal injection of sodium pentobarbital (50 mg/kg), and then infected intranasally with 2.4×10^6^ TCID_50_ RSV/A/long strain at week 5 after the first immunization. On day 3 or 5 post-challenge, mice were sacrificed and lungs were harvested for virus titer tests and immunological and histopathological analysis. Bodyweight was measured every day until mice were euthanized.

Animal studies were carried out in accordance with the Guide for the Care and Use of Laboratory Animals (National Research Council), and all animal procedures were reviewed and approved by the Animal Welfare and Research Ethics Committee at Jilin University.

### ELISA

RSV-F protein-specific antibodies (IgG, IgG1, and IgG2a) in sera from immunized mice were detected by enzyme-linked immunosorbent assay (ELISA). High-binding ELISA plates were coated with 0.25 μg/well RSV-F protein overnight at 4°C. Then, the plates were washed with PBS containing 0.05% Tween 20 three times and blocked with 1% bovine serum albumin dissolved in PBS for 1-2 h at 37°C. After removing the blocking buffer, serum samples serially diluted in 1% bovine serum albumin were added to the plate and incubated at 37°C for 1 h. Plates were then washed, followed by adding horseradish peroxidase (HRP) conjugated secondary antibody (Jackson ImmunoResearch, United States, 1:10,000 dilution) and incubated for 1 h at 37°C. Then, TMB was added to the plates at room temperature and the reaction was stopped with 2M H_2_SO_4_ after 20-30 minutes. Optical density (OD) was read at 450 nm in a microplate reader (Biotek, EL×800). For IgG isotyping, the ELISA test was performed as described above except that goat anti-mouse IgG1 or IgG2a antibody (Sigma-Aldrich, United States, 1:5,000 dilution) and anti-goat HRP-IgG (Jackson ImmunoResearch, United States, 1:10,000 dilution) were added to the plates and incubated at 37°C for 1 h, respectively, before development with TMB. Antibody titers were expressed as log 10 value of serum maximum dilution whose OD value was at least twofold higher than the average OD of the blank wells. The limit of detection (LOD) was 2. Any sample (such as PBS group) resulting in a titer less than the LOD was assigned a value of 1.52.

### Serum Neutralization Assay

Serum samples from mice to be tested were heat-inactivated at 56 °C for 30 minutes and two-fold serially diluted with the serum-free MEM medium. Similarly, the RSV/A/Long virus (ATCC, VR-26) was also diluted to 2 ×10^4^ TCID_50_/mL in the MEM medium. Subsequently, the diluted serum samples and virus were added to 96-well plates in equal volume (50 μL), mixed, and incubated for 2 h at 37°C. Moreover, each 96-well plate contained positive controls (virus only, no serum sample). After incubation, HEp-2 cells (ATCC, CCL-23) suspended in the growth medium were added to the serum-virus mixture in the number of 5 ×10^4^/well and incubated at 37°C. About one week later, the cells were fixed in 4% paraformaldehyde and stained with crystal violet. Stained plates were air-dried and evaluated by the cytopathic effect (CPE) using a dissecting microscope. Neutralizing antibody titers were assessed as described (14). The lowest dilution that resulted in 80% CPE inhibition was identified as the endpoint neutralizing antibody titer for that sample. The LOD was assigned as 4. Any sample with a titer less than the LOD was assigned a value of 2.

### Cytokine Detection in the Supernatant of Stimulated Splenocytes

At week 2 after boosting, spleens from immunized mice were cut into small fragments and pressed with the plunger seal of a 5 mL syringe to prepare a single-cell suspension. Red blood cells were then lysed with ammonium-chloride-potassium buffer, and splenocytes were washed with RPMI 1640 medium. 2 ×10^7^ cells were plated into 6-well plates and stimulated with 1 μg/mL RSV-F protein in the experimental groups or 1 μg/mL ConA in positive controls. After stimulation for 48 h, the culture supernatant was collected and centrifuged at 1,000 rpm for cytokine analysis. The secretion of IFN-γ, IL-2, IL-4, and IL-10 in the supernatant of stimulated splenocytes were quantified by mouse Th1/Th2 uncoated ELISA kits (ThermoFisher, USA). The plates were read at 450 nm in a microplate reader, and cytokine levels were expressed as pg/mL.

### IFN-γ ELISPOT

At week 2 after boosting, spleens were removed from immunized mice. After that, single-cell suspensions of splenocytes were prepared as described above. IFN-γ-secreting splenocytes were quantified using a mouse pre-coated IFN-γ ELISpot kit (Mabtech, Sweden) according to the manufacturer’s instructions. Briefly, the 96 well PVDF plates were washed 4 times with sterile PBS, then conditioned with RPMI 1640 medium containing 10% fetal bovine serum for at least 30 minutes at room temperature. After removing the medium, the splenocytes were seeded into 96-well plates at 10^6^/well and incubated with or without 1 μg/mL RSV-F protein for 24 h at 37°C. Cells incubated with PMA (50 ng/mL) and Ionomycin (1 μg/mL) were used as a positive control. The plates were emptied by discarding splenocytes and washed 5 times with PBS after incubation. The next steps were followed by incubation with the biotinylated anti-IFN-γ antibody for 2 h at room temperature, Streptavidin-HRP for 1 h, then ready-to-use TMB substrate solution. Deionized water was used to stop color development, and spot forming cells (SFC) were inspected and counted on an ELISpot reader (C.T.L, USA). The data were normalized to non-stimulated controls.

### Lung Virus Titers

On day 3 or 5 post-challenge, lungs were collected and homogenized in RPMI 1640 medium using glass tissue grinders. The clarified supernatants obtained by centrifugation were threefold serially diluted and inoculated with 2×10^4^/well HEp-2 cells (ATCC, CCL-23) in 96-well plates. After 5–7 days, wells with the CPE effect under the microscope were considered a positive result, or wells with detached cells caused by CPE after fixation and staining were deemed to be positive. The Reed-Muench method was used to calculate the TCID_50_ (33). The LOD was assigned as 9.

### Histopathology

Lung lobes were removed from mice on day 3 or 5 post-challenge and fixed with 4% paraformaldehyde for one week. Fixed lungs were embedded in paraffin blocks, sectioned, and stained with hematoxylin and eosin (H&E) or periodic acid–Schiff stain (PAS). H&E stained slides were observed with a light microscope (×200 magnification) and assessed using a semiquantitative scale based on peribronchiolar and bronchial infiltrates, perivascular leukocyte aggregates, according to previously described methods (34). Assigned score 0 is the surrounding space that is free or has few infiltrating cells, score 1 contains focal aggregates of infiltrating cells or the structure is cuffed by one definite layer of infiltrating cells, score 2 is cuffed by two defined layers of infiltrating cells and score 3 when the structure is cuffed by three or more definite layers of infiltrating cells with or without focal aggregates. PAS was used to identify goblet cell proliferation with airway mucus production. PAS stained slides were scored for goblet cells proliferation and mucus (1, none; 2, epithelial mucinous hyperplasia with none to rare luminal mucus; 3, epithelial mucinous hyperplasia with luminal mucus accumulation in airways; and 4, severe mucinous change with some airways completely obstructed by mucus) (35).

### Flow Cytometry

Mice were sacrificed on day 3 or 5 post-challenge and immediately perfused with sterile PBS through the right ventricle to eliminate the interference of cells in pulmonary vessels. Next, the lungs were excised, smashed, and enzymatically digested using the mouse lung dissociation kits (Miltenyi Biotec, Germany). Lung cells were blocked with CD16/32 antibody (BioLegend, Cat. No.156604) and stained on ice in the dark with surface antibodies, including PerCP-Cy5.5-CD3 (BD Bioscience, Cat. No. 560527), FITC-CD4 (BD Bioscience, Cat. No. 553650), APC-CD8 (BioLegend, Cat. No. 100712), and PE-CD69 (BioLegend, Cat. No. 104508). After staining, cells were acquired on a CytoFLEX flow cytometer (Beckman coulter) and analyzed using FlowJo software. The gating strategy is shown in **Supplementary Figure 3**.

### Statistical Analysis

The data were shown as mean ± standard deviation (SD). All statistical analysis was performed using GraphPad Prism software. The differences between two groups were analyzed using an unpaired t-test. Multiple comparisons were performed using one-way ANOVA or two-way ANOVA. The values were regarded as significantly different with P < 0.05 (*), P < 0.01 (**), P < 0.001 (***), P<0.0001(****).

## Results

### Effect of the Administration Routes on Immunogenicity of Various Adjuvanted RSV-F Vaccines

**Figure 2:**
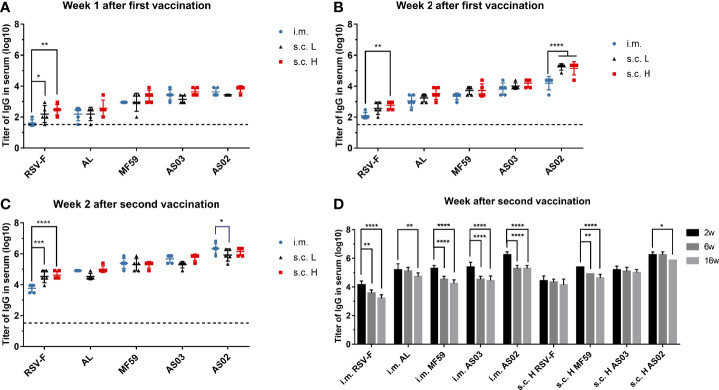
RSV-F-specific IgG titers in sera of mice intramuscularly or subcutaneously immunized with various RSV-F vaccines with or without adjuvants. The sera of mice in each group were detected at weeks 1 **(A)**, 2 **(B)** after the first vaccination, and weeks 2 **(C)**, 6 and 16 **(D)** after boosting by ELISA, respectively. Each data point represents an individual animal. Results were shown as mean ± SD of antibody titers calculated from five mice per group. The limit of detection (LOD) was 2. The dashed line with a value of 1.52 represented the results of PBS group. Data of panel **(A–D)** were collected from two independent experiments, respectively. *P< 0.05, **P <0.01, ***P< 0.001, ****P<0.0001 (two-way ANOVA with Tukey’s multiple comparisons test).

In order to investigate the effect of inoculation routes on the immunogenicity of different adjuvanted RSV-F vaccines, BALB/c mice were administrated intramuscularly or subcutaneously with RSV-F protein alone or adjuvanted RSV-F vaccines and sera were collected at different time points. All groups showed gradually increased body weight to varied degrees (**Supplementary Figure 1**), suggesting no systemic adverse events. The levels of RSV-F-specific IgG antibodies were measured by ELISA, and the results are shown in **Figure 2**. One week after the first immunization, mice in all groups produced detectable RSV-F-specific IgG antibodies (**Figure 2A**). With the same antigen dose, the RSV-F s.c. L group showed a higher binding antibody level than the RSV-F i.m. group, but all adjuvanted vaccines administered subcutaneously or intramuscularly induced comparable antibody levels. With the increasing antigen dose, antibody titers elicited by the high-dose antigen delivered subcutaneously were generally higher than that of the corresponding i.m. group, although not statistically significant. Two weeks after priming, all groups showed improved IgG titers (**Figure 2B**). With the same antigen dose, the antibody levels of all s.c. L groups were higher than those of i.m. groups. However, no statistically significant difference was found except for the AS02 groups. Regardless of inoculation routes and antigen dose, all groups gave an order of adjuvant activity: AS02>AS03, MF59>AL, which agrees well with our previous study (14). Two weeks after boosting, the antibody titers in all groups were greatly improved (**Figure 2C**). For the RSV-F group, the effect of administration routes and antigen dose on RSV-F-specific IgG antibody is similar to that one week after the first immunization. It was worth noting that, for each adjuvanted vaccine, the level of antibodies in i.m. group was slightly higher than that in s.c. L group and was comparable to that in s.c. H group. However, no statistically significant difference was found between them except for AS02 groups. Besides, the order of adjuvant activity of used adjuvants was unchanged. The above results indicated that subcutaneous inoculation induced faster and stronger IgG antibodies after the initial immunization, while intramuscular inoculation is more advantageous than subcutaneous injection after boosting as evidenced by the slightly higher antigen-specific antibody levels for adjuvanted RSV-F vaccines.

In terms of antibody persistence, IgG antibodies in sera from i.m. groups and the s.c. H groups except AL s.c H group were examined at weeks 2, 6, and 16 after boosting (**Figure 2D**). As expected, the antibody titers of all groups for each vaccine reached a peak at week 2 after boosting followed by different degrees of decrease. However, antibody levels of the i.m. groups declined faster than that of the corresponding s.c. H groups, suggesting antigen dose and/or inoculation routes may affect the persistence of the resultant immune response.

### Effect of the Administration Routes on Neutralizing Antibody Level of Various Adjuvanted RSV-F Vaccines

**Figure 3:**
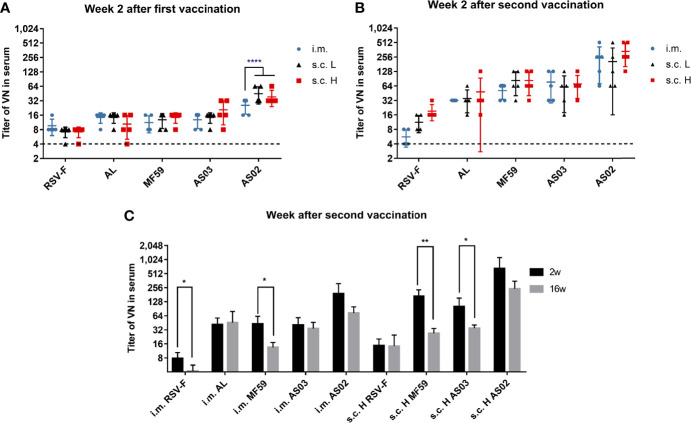
Serum neutralizing antibody titers in immunized mice. Sera from mice vaccinated with various RSV-F vaccines with or without adjuvants *via* intramuscular or subcutaneous injection were tested at week 2 after priming **(A)** or boosting **(B)**. Serum neutralizing antibody levels were also examined at weeks 2 and 16 after boosting in the long-term experiment **(C)**. Data shown was as mean ± SD of neutralizing antibody titers calculated from five mice in each group. The LOD was assigned as 4. The dashed lines represented the results of the PBS group with a value of 4. *P< 0.05, **P <0.01, ****P<0.0001 (two-way ANOVA with Tukey’s multiple comparisons test).

Besides antigen-specific antibody, the levels of neutralizing antibody in sera were also measured at week 2 after priming and boosting, and the results are shown in **Figure 3**. The order of neutralizing activity of adjuvanted vaccines showed similar trends to that of RSV-F-specific antibody titers regardless of injection routes and antigen dose: AS02>AS03, MF59 >AL. For each adjuvanted vaccine, there was no significant difference in neutralizing antibody titers between i.m., s.c. L and s.c. H groups except AS02 groups at week 2 after priming (**Figure 3A**). However, 2 weeks after boosting, each adjuvanted vaccine showed comparable neutralizing antibody levels regardless of antigen dose and inoculation routes (**Figure 3B**). Further investigation revealed that the neutralizing antibody levels peaked at week 2 after boosting followed by different degrees of decline in all groups except the AL group. However, even at week 16 after boosting, under the same conditions (immunization route and antigen dose), neutralizing antibody titers elicited by the AS02-adjuvanted vaccine still remained superior over those of AS03 and MF59-adjuvanted vaccines (**Figure 3C**). The above results indicate that adjuvant is a more important factor than inoculation route in eliciting long-lasting neutralizing antibodies. In addition, it is worth noting that low-dose RSV-F antigen delivered intramuscularly in presence of adjuvants can induce comparable neutralizing antibody level to that of a tenfold dose of antigen administered subcutaneously at week 2 after boosting, suggesting the antigen-sparing effect.

### Effect of the Administration Routes on Immune Response Types of Adjuvanted RSV-F Vaccines

**Figure 4:**
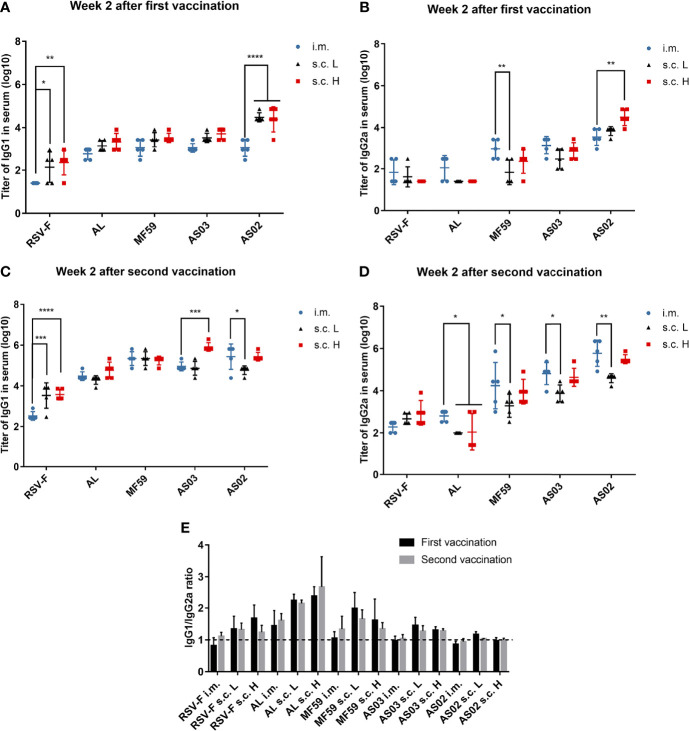
RSV-F-specific IgG1 and IgG2a antibodies and IgG1/IgG2a ratios. Sera from mice primed **(A, B)** and boosted **(C, D)** with various adjuvanted RSV-F vaccines or RSV-F alone were assessed by ELISA for their RSV-F-specific IgG1 and IgG2a antibodies. The ratios of IgG1 to IgG2a for each mouse were also calculated **(E)**. The dashed line indicated IgG1/IgG2a ratio= 1. Data represented the mean ± SD of each group (n=5). *P< 0.05, **P <0.01, ***P< 0.001, ****P<0.0001 (two-way ANOVA with Tukey’s multiple comparisons test).

An effective RSV vaccine usually requires both high levels of RSV-neutralizing antibody and Th1-like cellular immune response (36, 37). To explore the effect of immune routes on immune response types of adjuvanted RSV-F vaccines, the levels of IgG1 and IgG2a antibodies against RSV-F protein were determined at week 2 after the first and second immunizations, respectively (**Figure 4**). In BALB/c mice, the production of IgG2a is generally recognized as a characteristic of Th1 responses, whereas Th2 responses are associated with the production of IgG1 (38). At week 2 after the initial immunization, for all vaccines, the levels of IgG1 in the s.c. groups were higher than those in the i.m. groups (**Figure 4A**). Among them, the RSV-F group and AS02 group displayed significant differences. At week 2 after boosting, IgG1 levels in all groups increased (**Figure 4C**). However, the level of IgG1 elicited by adjuvanted vaccines *via* i.m. injection improved to comparable or higher levels when compared with that in the corresponding s.c. group, although no significant difference between the same adjuvanted vaccine except for AS02. In the case of IgG2a, as expected, aluminum adjuvant hardly induced IgG2a antibodies even after boosting (**Figures 4B, D**). However, all three squalene-based emulsion adjuvants induced improved IgG2a levels when compared with those of RSV-F alone and AL groups especially after boosting, regardless of inoculation routes and antigen dose. The level of IgG2a induced by AS02 was higher than that of AS03 in all cases, which was followed by MF59. Interestingly, each squalene-based vaccine administrated *via* i.m. route showed comparable or slightly higher IgG2a levels than that inoculated *via* s.c. route even with a higher antigen dose. The results of IgG1/IgG2a ratio indicated that boosting cannot change the bias of immune response, and that inoculation routes can influence immune type bias to varied degrees (**Figure 4E**). However, the adjuvant is a more critical factor to adjust the immune type bias, especially for potent adjuvant, such as AS02 that elicited a balanced Th1/Th2 immunity regardless of immunization routes (**Figure 4E**).

**Figure 5:**
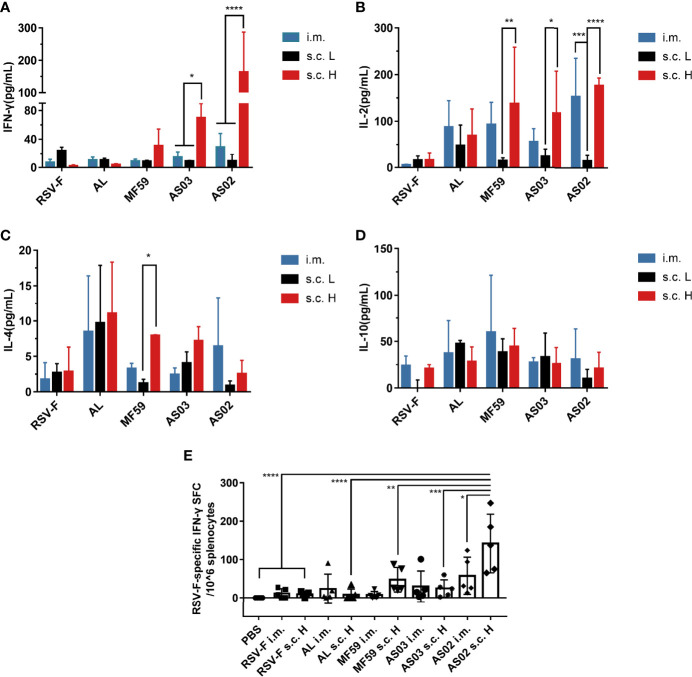
Cytokine levels in immunized mice. Spleen cells were collected from immunized mice at week 2 after boosting and restimulated with RSV-F protein. The secreted cytokines, including IFN-γ **(A)**, IL-2 **(B)**, IL-4 **(C)**, and IL-10 **(D)**, were measured from the supernatants using mouse Th1/Th2 uncoated ELISA kits. In addition, the number of RSV-F-specific IFN-γ secreting splenocytes was evaluated by ELISpot **(E)**. Data were collected from two independent experiments. Means ± SD of five mice per group was shown. *P< 0.05, **P <0.01, ***P< 0.001, ****P<0.0001 (two-way ANOVA with Tukey’s multiple comparisons test).

Besides IgG1 and IgG2a levels, cytokine levels produced by spleen cells stimulated *in vitro* were also evaluated using the ELISA assay. The examined cytokines include Th1 cytokines IFN-γ (**Figure 5A**) and IL-2 (**Figure 5B**), Th2 cytokine IL-4 (**Figure 5C**), and an immunomodulatory cytokine IL-10 (**Figure 5D**). The levels of the above four cytokines induced by the aluminum adjuvant group were not affected by either inoculation routes or antigen dose. As expected, aluminum adjuvant showed relatively higher IL-4 levels than other adjuvants regardless of administration route and antigen dose (**Figure 5C**). Interestingly, squalene-based emulsion adjuvants with high-dose RSV-F delivered subcutaneously elicited higher levels of IFN-γ, IL-2, and IL-4 than those of the corresponding low-dose groups, which may be accounted for the increased antigen dose. However, the IFN-γ and IL-2 levels in the i.m. group were higher to varying degrees than those in the corresponding subcutaneous injection group at the same antigen dose (**Figures 5A, B**), indicating that the intramuscular route could induce a more Th1-biased immunity when immunized with low doses of antigen. IL-10 is an immunomodulator that plays a crucial role in controlling disease severity in RSV infection (39, 40). However, the expression levels of IL-10 elicited by each adjuvanted vaccine did not show statistically significant differences regardless of the inoculation route and antigen dose (**Figure 5D**).

To further determine whether adaptive cellular immunity was enhanced, the number of RSV-F-specific IFN-γ secreting splenocytes was evaluated by ELISpot at week 2 after boosting (**Figure 5E**). As expected, aluminum adjuvant groups did not show an improved percentage of RSV-F-specific IFN-γ secreting splenocytes compared with that of RSV-F alone groups regardless of inoculation route and antigen dose. For MF59- and AS03-adjuvanted vaccines, varied increases in RSV-F-specific IFN-γ secreting splenocytes were found in comparison with those of the corresponding RSV-F alone group. However, there is no significant difference between different administration routes. In the case of AS02, the s.c. H group showed a significantly increased number of IFN-γ-secreting cells compared with that of intramuscular injection, which agrees well with the results of the ELISA assay.

### Challenge Test

**Figure 6:**
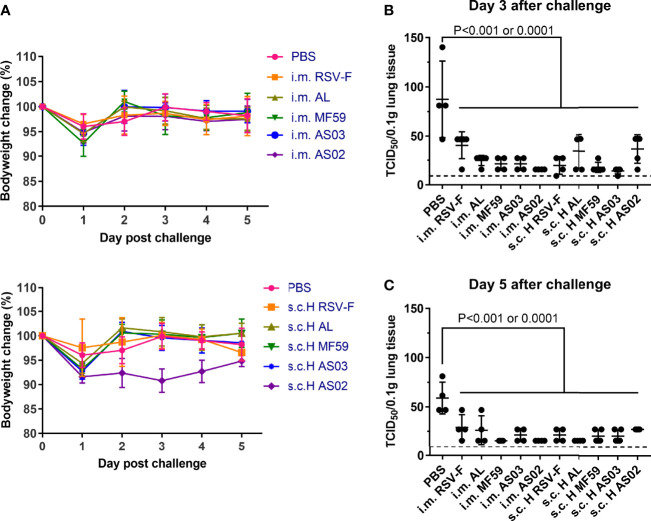
Mice body weight changes and lung virus titers after RSV challenge. Immunized mice (n= 8-10) were intranasally challenged with RSV/A/long at week 3 after boosting. **(A)** Bodyweight was monitored daily after the challenge until the mice were euthanized. **(B, C)** Lung virus titers were assessed by TCID_50_ assay on day 3 or 5 post-challenge. Each data point represented an individual animal. Dashed lines indicated limit of detection with a value of 9. Statistical significance was performed by two-way ANOVA with Tukey’s multiple comparisons test in GraphPad Prism. Groups that were significantly different from PBS group were marked with P-values.

To determine the efficacy of the abovementioned vaccine candidates, a virus challenge test was performed at week 5 after the first immunization. All groups showed bodyweight loss to varied degrees one day after challenge (**Figure 6A**). However, no statistically significant difference was found between them and PBS control group. In addition, all groups recovered to normal level on day 2 after challenge except the AS02 s.c. H group. The nearly 10% body weight loss of mice in AS02 s.c. H group remained for three days after challenge, and then gradually recovered to a comparable level to that of the PBS group on day 5 post-challenge, suggesting possible systemic deterioration after vaccination. Besides body weight, we also evaluated virus load in mouse lungs on days 3 and 5 after challenge (**Figures 6B, C**). On day 3 post-challenge, the PBS group showed the highest viral load, followed by the RSV-F group. Compared with PBS and RSV-F, the virus titers of other groups immunized with adjuvanted vaccines were basically equal to or slightly higher than the detection limit, suggesting effective virus clearance. On day 5 after challenge, RSV titers of all groups were comparable to or lower than the detection limit except PBS group.

**Figure 7:**
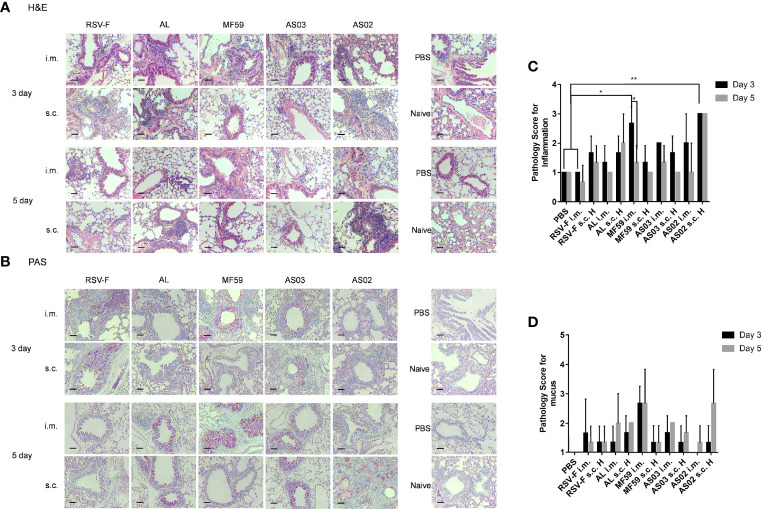
Histopathological changes of mouse lungs after RSV challenge. Lungs were collected from mice on day 3 or 5 post-challenge and tissue sections were stained with H&E **(A)** and PAS **(B)** to assess pulmonary histopathologic changes. Each panel (x200 magnification) represented an individual mouse from the indicated group. PBS, PBS control mice with RSV infection. Naïve, unimmunized and unchallenged mice. Scale bar: 200 μm. **(C)** Pathology score for inflammation. Inflammation responses on H&E stained tissue sections were scored on a scale of 0–3 according to diagnostic criteria (34). **(D)** Pathology score for mucus. PAS stained slides were scored for goblet cell proliferation and mucus on a scale of 1–4 (35). Results (n= 3 per group) were presented as mean ± SD and statistical significance was performed by two-way ANOVA with Tukey’s multiple comparisons test. *P< 0.05, **P <0.01.

The inflammation response in the lung was also detected by H&E and PAS analysis and relevant pathological scores were determined according to the scoring criteria (**Figure 7**). On day 3 after challenge, all groups inoculated with adjuvanted vaccines showed varied inflammation (**Figures 7A, C**). Among, mice inoculated with MF59 and AS03 adjuvants *via* the intramuscular route showed slightly more inflammatory cell infiltrations in the lungs than those *via* subcutaneous route, respectively. In contrast, mice immunized intramuscularly with RSV-F, AL, and AS02 alone showed relatively milder inflammation response than those subcutaneously, respectively. Fortunately, the lung inflammation of mice immunized with MF59- and AS03-adjuvanted vaccines or RSV-F alone alleviated from day 3 to 5 after challenge regardless of immunization routes. However, lung inflammation of mice in the AL s.c. H group and AS02 s.c. H group worsened instead, which was not observed in the i.m. groups. The results of mucus secretion showed roughly similar phenomena to the above inflammation responses (**Figures 7B, D**). As expected, aluminum adjuvant showed steady deterioration in mucus secretion from day 3 to 5 after challenge. In addition, both MF59 i.m. group and AS02 s.c. H group displayed more proliferated goblet cells and increased mucus secretion in the airway cavity on day 5 after challenge.

**Figure 8:**
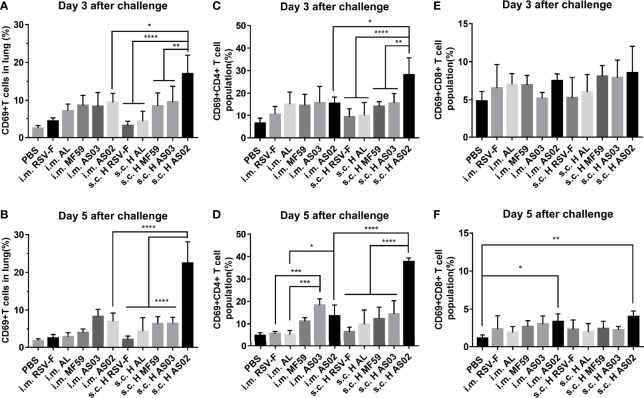
Activated T cells and their subsets in the lungs of immunized mice after challenge. On day 3 or 5 post-challenge, mice (n= 4 or 5) from different groups were euthanized and lungs were removed to analyze the expression levels of active T cells **(A, B)**, Th cells **(C, D)**, and Tc cells **(E, F)** through the expression of CD69 by flow cytometry. Data were analyzed with two-way ANOVA with Tukey’s multiple comparisons test. *P< 0.05, **P <0.01, ***P< 0.001, ****P<0.0001.

Besides histopathological changes in the lung, we further detected activated T cells and their subsets in the lung on day 3 or 5 post-challenge using flow cytometry (**Figure 8**). CD69 expression is readily upregulated upon activation in most leukocytes, which makes it a typical marker of activated lymphocytes (41). On day 3 after challenge, both intramuscular and subcutaneous groups inoculated with various vaccines gave the order of the proportion of activated T cells in the lung tissue of AS02> MF59, AS03>AL>RSV-F alone (**Figure 8A**). The significantly higher proportion of activated T cells in AS02 s.c. H group than that of AS02 i.m. group was probably due to the inoculation route, but not the antigen dose, as the significant difference was not found in other paired groups. The similar phenomena remained on day 5 after challenge (**Figure 8B**). The CD4^+^/CD8^+^ T cell subpopulation in activated T cells was further analyzed (**Figures 8C–F**). The results showed that more activated CD4^+^ T cells (Th cells) rather than CD8^+^ T cells (Tc cells) were detected in mouse lung tissues in all groups. It is worth noting that the AS02-adjuvanted vaccine administrated *via* subcutaneous route resulted in gradually increased CD4^+^ T cells, while other vaccines showed decreased trends from day 3 to 5 after challenge. The statistically significantly high-activated CD4^+^ T cells in the AS02 s.c. H group may at least partially be responsible for the bodyweight loss and lung pathological changes described above.

## Discussion

Antibody production is one of the most important parts of adaptive immune response and is closely related to the differentiation and maturity of B cells. At the early stage of vaccination or infection, naive B cells can quickly differentiate into moderate-affinity and short-lived plasmablasts through extrafollicular response in the spleen or the draining lymph nodes (42). The resulting plasmablasts are responsible for the production of the early protective antibodies, which usually function quickly and peak about day 5-7 after immunization or infection (43). In this study, with the same antigen dose, subcutaneous inoculation induced comparable IgG antibodies to that of intramuscular injection one week after the initial immunization regardless of adjuvants (**Figure 2A**). The result suggests that both routes probably own comparable capacity to promote the formation of plasmablasts for adjuvanted vaccines. It is well known that at the late stage of vaccination or infection, some activated B cells, including plasmablasts, may enter or re-enter the B cell follicle of the germinal center in the draining lymph nodes to differentiate into high-affinity and long-life plasma cells and memory B cells *via* follicular response (44). This process and the germinal center formation will take one or a few weeks (43). The resultant plasma cells can produce and sustain a high-level protective antibody secretion. In this study, at 2 weeks after the first vaccination, slightly higher IgG levels of the subcutaneous route (**Figure 2B**) were found, which should come from both plasmablasts and part of early plasma cells. Interestingly, there is no higher neutralizing antibody levels were observed for the subcutaneous route (**Figure 3A**). However, 2 weeks after boosting, intramuscular injection caused higher or comparable IgG antibody and neutralizing antibody levels even with the same antigen dose but half the dose of adjuvants for the subcutaneous route (**Figures 2C** and **3B**). The result indicates the superiority of intramuscular injection of adjuvanted RSV-F vaccines over the subcutaneous route in producing plasma cells. Moreover, RSV-F antigen formulated with adjuvants *via* intramuscular injection can elicit comparable or slightly higher IgG antibody and neutralizing antibody titers than those of a tenfold dose of antigen plus a twofold dose of adjuvant inoculated *via* subcutaneous route at week 2 post boosting. The dose-sparing effect may be valuable in practice. And the finding is basically in line with previous reports that intramuscular inoculation has better or equal immunogenicity than subcutaneous injection because of the better vascularization of muscle, and adjuvanted vaccines are more suitable for intramuscular injection (23, 25). In comparison with the inoculation route, the adjuvant is more critical in regulating the generation of both plasmablasts and plasma cells and therefore the production of IgG and neutralizing antibodies. It is supported by the data of antibody persistence and the fact that both vaccination routes showed consistent performance in terms of adjuvant potency at various sampling time points (AS02>AS03, MF59>AL) (**Figures 2** and **3**).

Besides antibody, Th1-biased cellular immune response is preferred for developing the RSV vaccine (36, 37). It is well known that after activation, naïve CD4^+^ T cells can polarize into Th1, Th2, Th17, T regulatory, and T follicular helper cell subsets *via* dendritic cell-mediated differentiation in draining lymph nodes (45). The type of cell subset is mainly determined by cytokines secreted by migrated dendritic cells and other innate immune cells in the draining lymph node microenvironment. The resulting Th1 and Th2 cell subsets can further secret distinct cytokines to regulate cellular and humoral immune responses, respectively (46). In particular, the differentiation of Th1 cells involves IL-12 and IFN-γ, while Th2 cells can be induced under the microenvironment dominated by IL-4. And Th1 cells can further secret the “classical” Th1 cytokines TNF, IFN-γ, and IL-2, and Th2 cells usually produce IL-4, IL-5, and IL-13 (47). In this study, the results of cytokine secretion from stimulated splenocytes indicate that with the same antigen dose, the intramuscular route is probably more supportive in eliciting a Th1-biased immune response than the subcutaneous route regardless of adjuvant type. However, compared to the inoculation route, the adjuvant is a more decisive factor in mediating immune type bias (**Figure 5**). Among all adjuvants, squalene-based emulsion adjuvants tend to induce a balanced Th1/Th2 immune response *via* the intramuscular route, while only AS02 remains the same tendency when inoculated *via* subcutaneous route as evidenced by the similar IgG1/IgG2a ratios (**Figure 4**). The superior performance of AS02 delivered intramuscularly has also been observed in virus clearance capacity after the challenge (**Figures 6B, C**).

Although the above advantages resulted from AS02, we found that it led to serious adverse effects at the local injection site (**Supplementary Figure 2**) and significant body weight loss (**Figure 6A**) when inoculated subcutaneously. We speculate that the above adverse effects may come from prolonged retention of AS02 adjuvant at the local subcutaneous tissue after injection (48). Unfortunately, severe lung lesions have also been observed for AS02 s.c. H group (**Figure 7**) after the challenge. However, no similar phenomena were found when the AS02-adjuvanted vaccine was injected intramuscularly. To illustrate the mechanism, we detected the percentage of activated T cells and their subtypes and found that more CD4^+^ T cells rather than CD8^+^ T cells were activated in mouse lung tissues in the AS02 s.c. H group and the trend was even more apparent on day 5 post-challenge (**Figure 8**). This finding is important for practice because it is generally accepted that excessive activated CD4^+^ T cells could contribute to enhanced respiratory disease (ERD) development (49). That no severe side effects happened in both local and system for the intramuscular route is probably because it is more efficient to antigen presenting and adjuvant delivery to draining lymph nodes (7, 50). Although the moderate local inflammatory reaction is usually indispensable to efficient immune cell recruitment and adequate antigen presentation, excessive inflammation can cause severe tissue damage, which may involve both innate and adaptive immune cells. However, the relationship between local excessive inflammatory reactions and abolished lung tissue protection after the challenge is still unclear. Further investigation is needed to illustrate the relevant mechanism. In practice, decreased adjuvant dose or alternative inoculation route should be considered.

**Figure 9:**
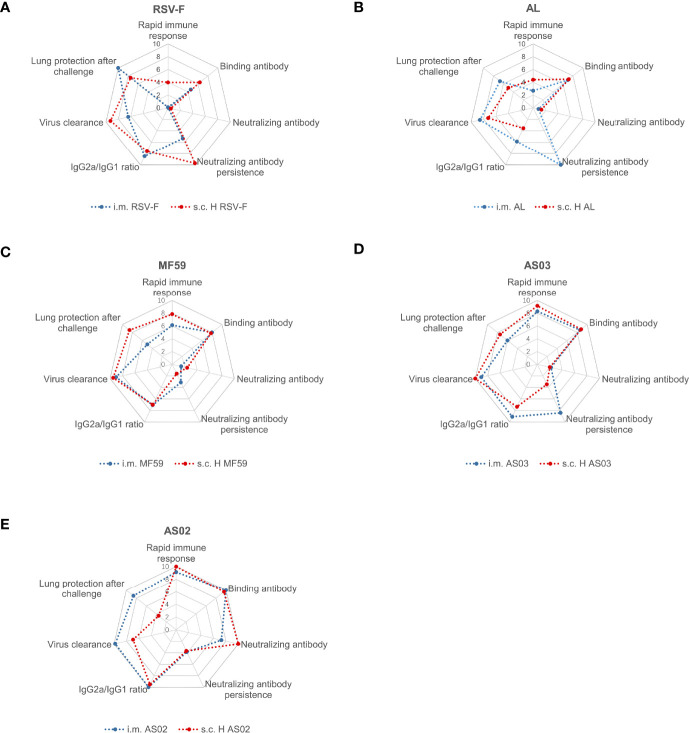
Radar chart of RSV-F vaccines with or without adjuvants *via* intramuscular and subcutaneous routes. RSV-F alone **(A)**, Alhydrogel-adjuvanted vaccine **(B)**, MF59-adjuvanted vaccine **(C)**, AS03-adjuvanted vaccine **(D)**, and AS02-adjuvanted vaccine **(E)**. The radar charts were defined as seven dimensions, namely “Rapid immune response” (D1), “Binding antibody” (D2), “Neutralizing antibody” (D3), “Neutralizing antibody persistence” (D4), “IgG2a/IgG1 ratio” (D5), “Virus clearance” (D6) and “Lung protection after challenge” (D7) dimension. The evaluation indexes Di was normalized with the method in the supplementary material, and integrated into the radar chart by the software of Excel. Within each chart, different axes represented different evaluation indexes that shared the same metric scale (0–10), and the value increased with distance from the center.

In order to illustrate the different properties of adjuvants used in this study, the data of antigen-specific antibodies, neutralizing antibodies, antibody subtypes, cytokines, immune response persistence, virus clearance, and lung protection after challenge were processed, normalized, and visualized with radar charts (**Figure 9**) according to the Method described in the Supplementary Material. MF59 and AS03 show similar characteristics in both inoculate routes (**Figures 9C, D**), while intramuscular injection of AS02-adjuvanted RSV-F subunit vaccine is more promising to maximize beneficial immune response and minimize undesired reactogenicity of vaccines (**Figure 9E**). Further optimization, such as adjuvant dose, antigen dose, and inoculation interval, is needed to develop a safe and efficient RSV-F subunit vaccine.

## Conclusion

In summary, this study investigated the effects of intramuscular and subcutaneous routes on the immune response and protective efficacy of various adjuvanted RSV-F vaccines (Alhydrogel-, MF59-, AS03- and AS02-adjuvanted vaccines). Evidence is presented that intramuscular inoculation is more advantageous in enhancing the immunogenicity of adjuvanted RSV vaccines after boosting when compared with subcutaneous inoculation, although subcutaneous injection can induce a quicker and higher immune response after the initial vaccination. In addition, the intramuscular route is safer than the subcutaneous route especially when a potent adjuvant, such as AS02, is formulated with antigen. Moreover, adjuvant, but not inoculation route, is a more decisive factor to regulate antibody production and immune response bias. In the future, the spectrum of cytokine expression, the persistence of the pulmonary inflammatory and its possible influence on lung function after challenge will be helpful to further illustrate different performance of the above various RSV-F vaccines after challenge. Moreover, further research is needed to examine RSV-F-specific cellular immune responses, the functional subsets of CD4+ T cells and tissue-resident memory T cells in lungs before and after challenge to explore the protective mechanism of adjuvanted RSV-F vaccines. Despite the above limitations, our findings provide valuable information about administration routes and adjuvants in the design and clinical transformation of novel RSV-F subunit vaccines.

## Data Availability Statement

The original contributions presented in the study are included in the article/**Supplementary Material**. Further inquiries can be directed to the corresponding author.

## Ethics Statement

The animal study was reviewed and approved by the Animal Welfare and Research Ethics Committee at Jilin University.

## Author Contributions

LJB, YZe, XHG, DDL, JYZ, LYJ, YC, JCL, and KZ carried out the experiments and collected the data. LJB and YZe performed the data analysis. LJB wrote the first draft of the manuscript. CLJ, WK, and YZa are responsible for the supervision and project administration. CLJ, WK, and YZa designed the study and contributed to the interpretation of the results. YZa revised the first draft of the manuscript. All authors discussed the results, commented on the manuscript, and approved the final article. All authors attest that they meet the ICMJE criteria for authorship.

## Funding

We are grateful for the support from the Scientific Research Foundation of the Education Department of Jilin Province, China (grant number JJKH20211058KJ), and the Jilin Province Science and Technology Development Plan (grant number 20200708085YY). This work was partly supported by a scholarship from the China Scholarship Council (CSC number 201906175182). Funding sources had no involvement in study design, in the collection, analysis, and interpretation of data, or in the writing of the report.

## Conflict of Interest

The authors declare the following financial interests/personal relationships which may be considered as potential competing interests: JCL was the employee of Changchun BCHT Biotechnology Co. CLJ and WK are both the faculty of Jilin University and employees of Changchun BCHT Biotechnology Co.

The remaining authors declare that the research was conducted in the absence of any commercial or financial relationships that could be constructed as a potential conflict of interest.

## Publisher’s Note

All claims expressed in this article are solely those of the authors and do not necessarily represent those of their affiliated organizations, or those of the publisher, the editors and the reviewers. Any product that may be evaluated in this article, or claim that may be made by its manufacturer, is not guaranteed or endorsed by the publisher.
