# Supplementary material for: Intramuscular Inoculation of AS02-Adjuvanted Respiratory Syncytial Virus (RSV) F Subunit Vaccine Shows Better Efficiency and Safety Than Subcutaneous Inoculation in BALB/c Mice
Source: Front Immunol. 2022 Jul 22;13:938598. doi: 10.3389/fimmu.2022.938598 (PMC9354885; doi:10.3389/fimmu.2022.938598)
Supplement: Supplementary file 1 [file DataSheet_1.docx]

Supplementary Material

# Method

## Data Processing and Normalization of Evaluation Index

| Dimension | Evaluation Index | Data Processing in each group |
| --- | --- | --- |
| D1 | Rapid immune response | Mean (M1) of RSV-F specific IgG antibody level at week 1 after priming |
| D2 | Binding antibody | Mean (M2) of RSV-F specific IgG antibody level at week 2 after boosting |
| D3 | Neutralizing antibody | Mean (M3) of neutralizing antibody level at week 2 after boosting |
| D4 | Neutralizing antibody persistence | Ratio (M4) of the mean titer of neutralizing antibody between week 16 and week 2 after boosting |
| D5 | IgG2a/IgG1 ratio | Mean (M5) of IgG2a/IgG1 ratio at week 2 after boosting |
| D6 | Virus clearance | The proportion (M6) of lung virus clearance on day 3 after challenge  (Average viral load in PBS group - average viral load in the experimental group) / average viral load in PBS group |
| D7 | Lung protection after challenge | The reciprocal (M7) of the mean sum of lung inflammation and mucus pathology scores for each group of mice on day 5 after challenge |

The above processed data was then normalized to be distributed in the range 0-10 using

$$Di = (Mi - Mi(min))/ (\mathrm{Mi}(max)-Mi(min))*10$$

Where Mi is the mean of evaluation index Di in an experimental group; Mi_(min)_ denotes the mean value of PBS group in D1, D2, D3 and D6 dimensions respectively, or value 0 in D4, D5 and D7 dimensions; and Mi_(max)_ represents the maximum mean value of evaluation index Di in all experimental groups.

# Supplementary Figures and Tables

## Supplementary Figures


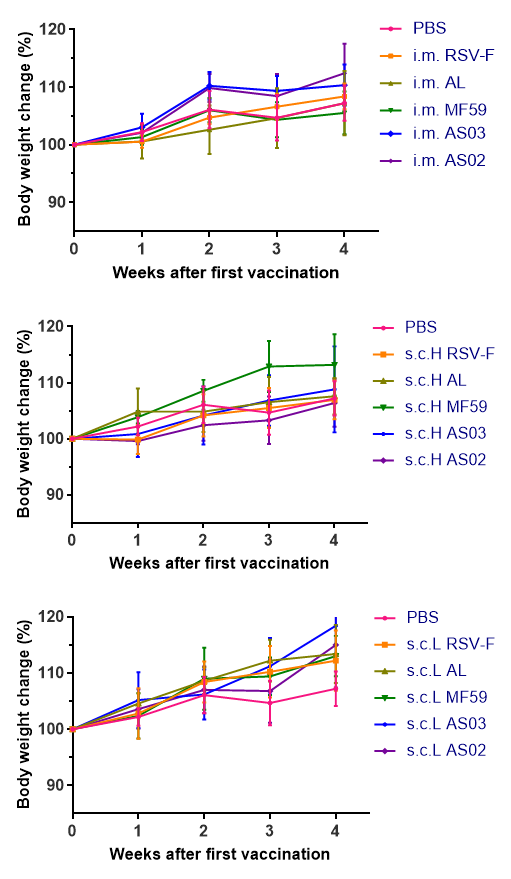


**Supplementary Figure 1.** Body weight change after the first vaccination. Groups of mice (n=5) were intramuscularly or subcutaneously immunized twice with various RSV-F vaccines with or without adjuvants at two-week interval. Body weights were measured every week after the first immunization. Data are shown as mean ± SD.


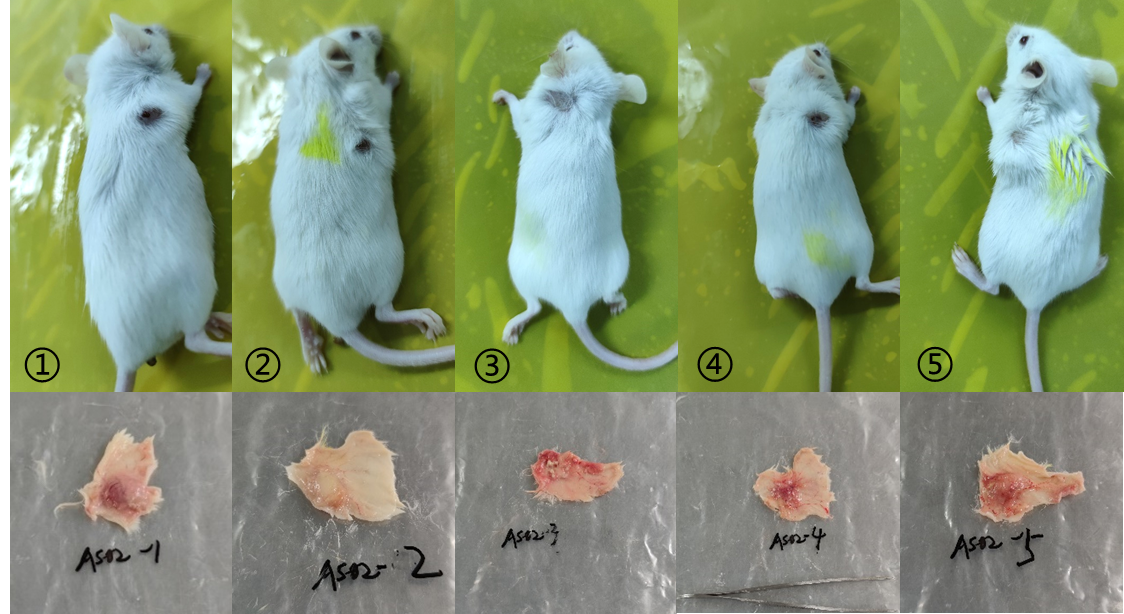


**Supplementary Figure 2.** The adverse effect at the local injection site of mice in the AS02 s.c. H group. Abscesses at the injection site appeared on day 2 or 3 after priming when mice were immunized subcutaneously with AS02 adjuvanted vaccines and disappeared three weeks after the second immunization. This side effect was commonly observed in the AS02 s.c. H group and the AS02 s.c. L group (data was not shown) after vaccination.

**Supplementary Figure 3.** Gating strategy for analyzing CD69+ T cells, CD69+ CD4+ T cells, and CD69+ CD8+ T cells in mouse lungs using flow cytometry. First, single cells within loose gates were gated to exclude doublets and debris. Next, the lymphocytes were identified and gated by their forward and side scatter. Cells expressing CD69 and CD3 molecules were considered activated T cells. The expression of CD69 CD4 or CD69 CD8 to analyze active T cell subtypes were then determined on CD3 positive events.
